# New Insights into Macrophage Polarization and its Prognostic Role in Patients with Colorectal Cancer Liver Metastasis

**DOI:** 10.21203/rs.3.rs-3439308/v1

**Published:** 2023-10-19

**Authors:** Isha Khanduri, Harufumi Maki, Anuj Verma, Riham Katkhuda, Gayathri Anandappa, Renganayaki Pandurengan, Shanyu Zhang, Alicia Mejia, Zhimin Tong, Luisa M. Solis Soto, Akshaya Jadhav, Ignacio I. Wistuba, Scott Kopetz, Edwin R. Parra, Jean-Nicolas Vauthey, Dipen M. Maru

**Affiliations:** The University of Texas MD Anderson Cancer Center; The University of Texas MD Anderson Cancer Center; Yale-New Haven Hospital; The University of Chicago Medical Center; The University of Texas MD Anderson Cancer Center; The University of Texas MD Anderson Cancer Center; The University of Texas MD Anderson Cancer Center; The University of Texas MD Anderson Cancer Center; The University of Texas MD Anderson Cancer Center; The University of Texas MD Anderson Cancer Center; The University of Texas MD Anderson Cancer Center; The University of Texas MD Anderson Cancer Center; The University of Texas MD Anderson Cancer Center; The University of Texas MD Anderson Cancer Center; The University of Texas MD Anderson Cancer Center; The University of Texas MD Anderson Cancer Center

**Keywords:** macrophage, polarization, liver metastasis, colorectal, multiplex, immunofluorescence, survival

## Abstract

**Background:**

As liver metastasis is the most common cause of mortality in patients with colorectal cancer, studying colorectal cancer liver metastasis (CLM) microenvironment is essential for improved understanding of tumor biology and to identify novel therapeutic targets.

**Methods:**

We used multiplex immunofluorescence platform to study tumor associated macrophage (TAM) polarization and adaptive T cell subtypes in tumor samples from 105 CLM patients (49 without and 56 with preoperative chemotherapy).

**Results:**

CLM exhibited M2 macrophage polarization, and helper T cells were the prevalent adaptive T cell subtype. The density of total, M2 and TGFβ-expressing macrophages, and regulatory T cells was lower in CLM treated with preoperative chemotherapy. CLM with right-sided primary demonstrated enrichment of TGFβ-expressing macrophages, and with left-sided primary had higher densities of helper and cytotoxic T cells. In multivariate analysis, high density of M2 macrophages correlated with longer recurrence-free survival (RFS) in the entire cohort [hazard ratio (HR) 0.425, 95% CI 0.219–0.825, p=0.011) and in patients without preoperative chemotherapy (HR 0.45, 95% CI 0.221–0.932, p=0.032). High pSMAD3-expressing macrophages were associated with shorter RFS in CLM after preoperative chemotherapy.

**Conclusions:**

Our results highlight the significance of a multi-marker approach to define the macrophage subtypes and identify M2 macrophages as a predictor of favorable prognosis in CLM.

## Introduction

Colorectal cancer is the third most common malignancy in the world and the second leading cause of cancer deaths globally (1). The incidence of colorectal cancer has been declining in North America, Australia, and Europe but increasing in parts of Asia and South America. Colorectal cancer incidence in individuals younger than 50 years of age is increasing (2). Liver is the most common non-regional site of colorectal cancer metastasis, and approximately 50% of patients with colorectal cancer develop liver metastasis (3, 4). Surgical resection is the only potentially curative treatment for colorectal cancer liver metastases (CLM) (5, 6). Oxaliplatin- or irinotecan-based systemic chemotherapy is used in combination with targeted agents (anti-vascular endothelial growth factor and epidermal growth factor inhibitor) for perioperative treatment of CLM (7). However, more than 50% of patients with CLM develop recurrence after resection, most often within 2 years (8, 9). In patients with CLM, clinical outcomes and therapeutic responsiveness are determined in part by tumor somatic mutation status, especially RAS mutations, which predict higher relapse rates and poor surgical outcomes (10–12).

In addition to genetic makeup of the tumor cells, the tumor microenvironment (TME) is critical in determining response to chemotherapy, immunotherapy, and other targeted therapies (13). Tumor-associated macrophages (TAMs) form a major component of the TME (14) and demonstrate significant plasticity. Two major activation/polarization states in response to environmental stimuli have been described for macrophages. Classically activated macrophages, referred to as M1 macrophages, are activated by cytokines like interferon-gamma, tumor necrosis factor-1, and lipopolysaccharides. Alternatively activated macrophages, referred to as M2 macrophages, are induced by interleukin-4, interleukin-13, and transforming growth factor beta (TGFβ) (15–17).

The prognostic significance of TAMs in primary colorectal cancers has been extensively studied; however, the complexity of TAMs and their influence on survival of patients with CLM has not yet been determined. To address this gap in knowledge, we used multiplex immunofluorescence tyramide signal amplification to quantify and determine the polarization of macrophages in CLM, and we examined the relationship between TAMs and recurrence-free survival (RFS). We identified CD68 + CD163 + M2 macrophages as a predictor of better RFS.

## Materials and methods

The study was approved by the Institutional Review Board of The University of Texas MD Anderson Cancer Center with a waiver of informed consent (protocol no: LAB-09-0373).

### Patient population and samples

Patient eligibility criteria included resection of liver metastasis of colorectal adenocarcinoma during 2002–2007, completion of hepatic resection with intent to resect all CLM (23), and the presence of viable tumor in the resection specimen. Pathologic response was graded based on criteria described previously (21, 22). Patients who underwent macroscopically incomplete resection (R2 resection) or hepatectomy for recurrent CLM and patients with complete pathologic response to preoperative chemotherapy were excluded from the study.

The study included 105 patients with liver metastasis of colorectal adenocarcinoma who underwent liver resection with (n = 56) or without (n = 49) preoperative chemotherapy. Patients’ demographic, clinical, and pathologic characteristics, type of preoperative chemotherapy, and pathologic response to chemotherapy were retrieved from the electronic medical records. Tumor (pT) category of the primary colorectal cancer was classified according to the *AJCC Cancer Staging Manual,* eighth edition.

### Processing of surgically resected colorectal liver metastases

Surgically resected samples from all the patients were reviewed to determine tumor viability and adequacy for construction of tissue microarrays. Two tissue microarrays composed of 203 cores from 105 patients (average 1.9 cores/tumor) were constructed by obtaining one or two 1-mm^2^ cores from formalin-fixed, paraffin-embedded blocks of each CLM. The cores were obtained from two nonnecrotic and nonadjacent regions of the tumor nodule. The entire area of each tissue core was subjected to multiplex immunofluorescence phenotyping.

### Multiplex immunofluorescence phenotyping

Macrophages have been widely studied in solid tumors using the general macrophage marker CD68 or other single markers to detect M1 and M2 macrophage polarization (29–32). However, due to recent developments and insights into macrophage polarization, TAMs and their subtypes are better characterized by assessing co-expression of multiple markers by multiplex analytical platforms like multiplex immunofluorescence. Common markers for M1 macrophages are CD86, CD11c, HLA-DR, inducible nitric oxide synthase, and MRP8-14, and for M2 macrophages are CD163, CD206, CD204, and Arginase 1 (33). We studied independent expression (expression of a single marker without quantitating any co-expression) and co-localization of CD68, CD163, CD206, CD86, Arginase 1, and MRP8-14, along with T cell markers (CD3, CD4, CD8 and FOXP3), using multiplex immunophenotyping. This approach evaluates the impact of co-expression of markers on quantification of the macrophage subtypes (Supplementary Table S1). M1 macrophage subtype was identified by co-expression of CD68 and CD86 and absence of expression of CD163, CD206, and Arginase 1. M2 macrophage subtype was identified by co-expression of CD68 and CD163 and absence of expression of CD86 and MRP8-14. Additionally, expression of TGFβ and pSMAD3 on tumor cells, TAMs, and adaptive T cells was analyzed, along with the impact of such expression on survival of patients with CLM.

Automated multiplex immunofluorescence staining was performed on 4-micrometer-thick formalin-fixed, paraffin-embedded tissue microarray sections using techniques developed and validated previously (24–26). The stained slides were scanned using Vectra Polaris 3.0.3, a multispectral imaging system (Akoya Biosciences, Marlborough, MA, USA)), at a 200x magnification.

The immunofluorescence markers were grouped into two panels for analysis of the macrophage and T cell populations: panel 1 included common macrophage markers and markers for macrophage polarization; CD68, CD163, CD86, CD206, MRP8-14, Arginase 1, and epithelial marker (pan cytokeratin), and panel 2 included markers for macrophages, T cell subtypes, TGF-B and Psmad3 expression on macrophage and T cells; CD68, CD3, CD4, CD8, FOXP3, pSMAD3, TGFβ, and epithelial marker (pan cytokeratin) (Supplementary Table S2).

### Multispectral analysis

**Figure 1:**
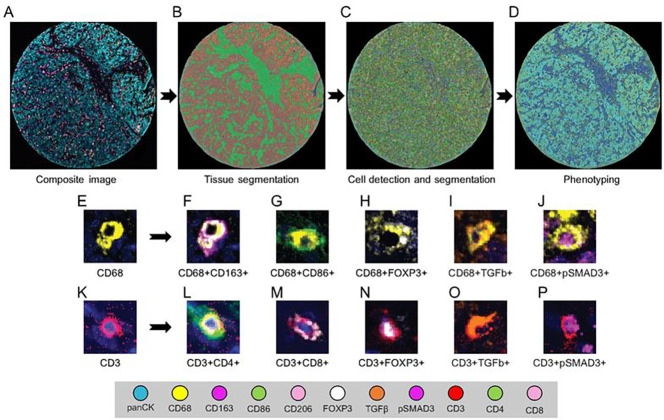
Workflow of multiplex immunofluorescence digital image analysis. **A,** Multiplex immunofluorescence image of a representative tumor core. **B,** Tissue segmentation was performed by training the software using representative examples from each compartment (red-tumor, green-stroma). **C,** Cell limits were defined, and cells were individually identified. **D,** Cells were phenotyped based on expression of surface proteins. **E-J,** Representative examples of macrophages (E) and their specific phenotypes (F-J) based on co-expression of markers. **K-P,** Representative examples of adaptive T cells (K) and their specific phenotypes (L-P) based on co-expression of markers.

Inform 2.4.6 Image Analysis software (Akoya Biosciences, Marlborough, MA, USA) was used to analyze the scanned multispectral component images. The raw images were prepared by eliminating the autofluorescence emitted. Each region of interest was segmented into the epithelial component, composed of glandular structures and nests of malignant cells, and the stromal component, composed of the fibrous connective tissue intervening between the malignant cell clusters. Following tissue segmentation, individual cell segmentation was performed. This entailed identification and segmentation of the DAPI-stained cells using multiple parameters, including DAPI intensity, minimum nuclear size, splitting sensitivity, and cytoplasmic thickness. The images were then subjected to the Inform active phenotyping algorithm, which allows identification of individual cells based on their pattern of fluorophore expression and indicates the phenotype. Phenotypes were defined based on the markers present in the panel, and cells not expressing any of the markers were classified as “other.” The final phenotype of each cell was defined based on co-localization of antibodies (Supplementary Table S1), obtained by using the specific x and y spatial coordinates of each cell. In the final report, cell density was expressed as number of cells per square millimeter. Figure 1 shows the workflow of multiplex immunofluorescence digital image analysis.

### Statistical analysis

To evaluate the densities of biomarkers and association of cell phenotype distribution with survival, we dichotomized biomarker densities by the median. The differences in nonparametric continuous variables were assessed using the Mann Whitney U test. RFS was calculated from the date of liver resection to the date of recurrence. Survival curves were obtained using the Kaplan-Meier method and compared using the log-rank test. Multivariate Cox proportional hazards model analysis was performed including factors with a threshold p value less than 0.10 in univariate analyses for the final model (27, 28). All statistical tests were two-sided, and statistical significance was defined as a p value of less than 0.05. Statistical analysis was conducted with SPSS version 26.0 (SPSS Inc., Chicago, IL, USA) and GraphPad Prism 9.0.0 (GraphPad Software Inc., San Diego, CA, USA).

## Results

### Characteristics of the study population

Table 1 shows the clinicopathologic characteristics of the study population. The majority of the patients had left-sided primary tumors, lymph-node-positive primary tumors, and no evidence of extrahepatic metastasis.

Patients who received preoperative chemotherapy (n = 56; 53%) were older, had a higher frequency of synchronous CLM (p = 0.001), and had a higher number of CLM (p = 0.009); patients who did not receive preoperative chemotherapy (n = 49; 47%) had higher median carcinoembryonic antigen levels at diagnosis of CLM (p = 0.03).

### Density of macrophage and adaptive T cell subtypes in CLM:

**Figure 2:**
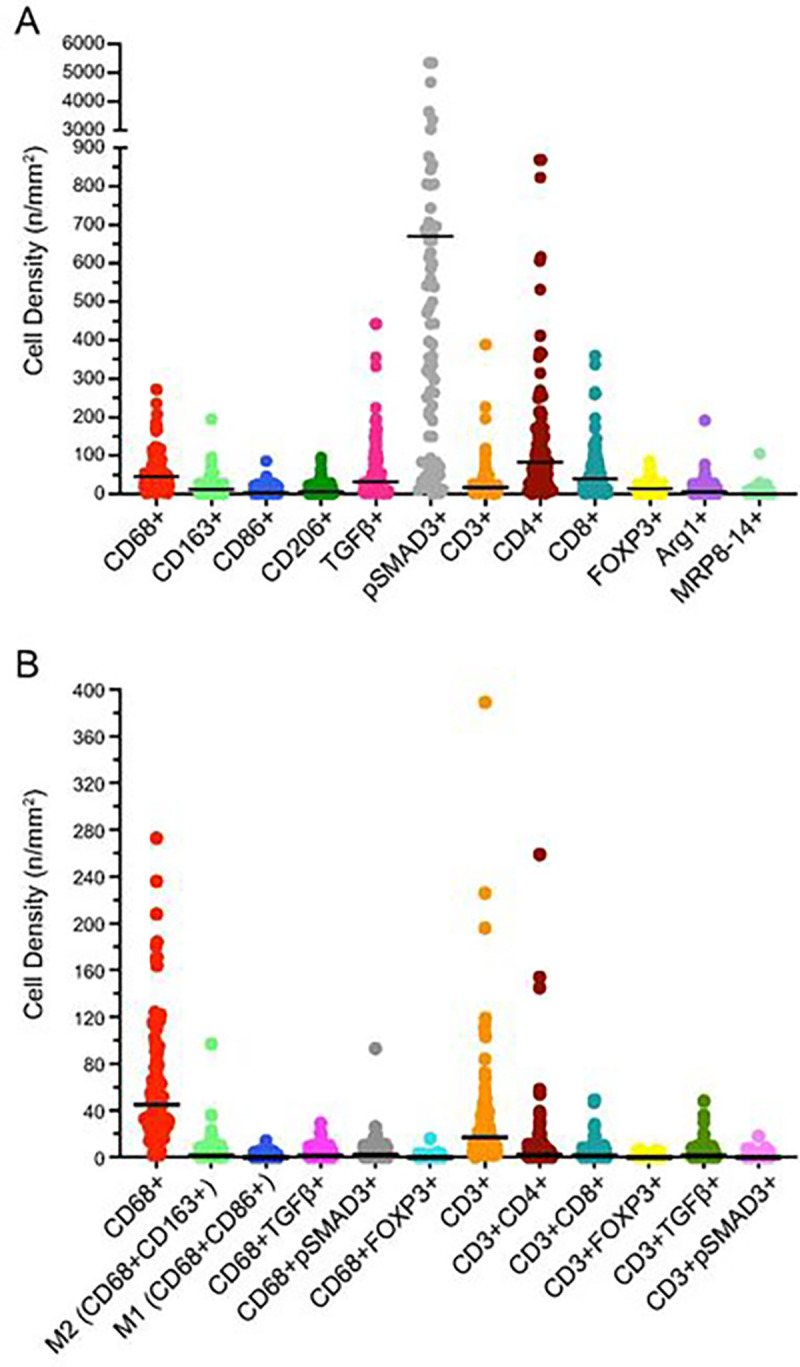
Density of different immune cell types. **A,** Density of cells expressing individual markers in the multiplex immunofluorescence panel. **B,** Density of macrophage and adaptive T cell subtypes defined by marker co-expression. The horizontal bars indicate median values.

Fig. 2A and Supplementary table 3 shows the cell density for the various markers used as part of the multiplex immunofluorescence panel for characterization of TAMs and adaptive T cells. The density of CD68 + macrophages was significantly higher than the density of CD3 + T cells [median (range), 45 (2–273) cells/mm^2^ vs 17 (0–389) cells/mm^2^; p = 0.0001]. pSMAD3 was the most abundant of the markers (median density, 660 cells/mm^2^) due to its high expression on tumor cells. CD4 had the highest cell density among the adaptive T cell markers [median (range) density, 81.29 (0–868.79) cells/mm^2^], likely due to its expression on both T cells and macrophages. Expression of TGFβ on tumor cells resulted in higher density of cells expressing TGFβ as compared to the density of cells co-expressing CD3 and TGFβ or CD68 and TGFβ (Fig. 2).

Among the TAMs, median (range) cell density was significantly higher for CD68 + CD163 + M2 macrophages than for CD68 + CD86 + M1 macrophages [1 (0–97) cells/mm^2^ vs 0 (0–14) cells/mm^2^, p = 0.001] (Fig. 2B, Supplementary Table S4). Hence, TAMs in our cohort exhibited M2 macrophage polarization. Median (range) cell density was significantly lower for TGFβ-expressing macrophages and pSMAD3-expressing macrophages [1 (0–29) cells/mm^2^ and 2 (0–93) cells/mm^2^, respectively, Supplementary Table S4] than for TGFβ-expressing tumor cells and pSMAD3-expressing tumor cells [4 (0–95) and 524 (0–4723), respectively, p < 0.01]. Median (range) cell density for FOXP3-expressing macrophages was very low: 0 (0–16) cells/mm^2^.

Of the adaptive T cells, helper T cells were the most abundant, with a median (range) density of 2 (0–259) cells/mm^2^ (Supplementary Table S3). Median (range) densities of cytotoxic T cells [1 (0–49) cells/mm^2^] and T regulatory cells [0 (0–6) cells/mm^2^] were relatively low. T cells overall (Fig. 2B, supplementary table 4) and the T cell subtypes (data not shown) exhibited absent to minimal TGFβ and pSMAD3 expression.

### Correlation of density of macrophage and adaptive T cell subtypes in CLM with clinicopathologic characteristics

A number of correlations were observed between macrophages and T cell phenotypes and clinicopathologic characteristics. The density of CD68 + CD163 + M2 macrophages was higher in CLM with largest diameter more than 3 cm than in smaller CLM. The density of TGFβ-expressing macrophages was higher in CLM with right-sided primary tumors (Table 2). The densities of helper T cells and cytotoxic T cells were higher in CLM with left-sided primary tumors. The density of pSMAD3-expressing cytotoxic T cells was higher in CLM with node-positive primary tumors. Densities of a number of cell phenotypes were higher in CLM not treated (vs treated) with preoperative chemotherapy: total macrophages, TGFβ-expressing macrophages, FOXP3-expressing macrophages, CD68 + CD163 + M2 macrophages, and pSMAD3-expressing helper T cells (Table 2). The densities of macrophage and adaptive T cell phenotypes did not differ by preoperative CEA level, timing of detection of CLM (synchronous or metachronous), or pathologic response to preoperative chemotherapy.

### Association of macrophage subtypes with RFS

**Figure 3:**
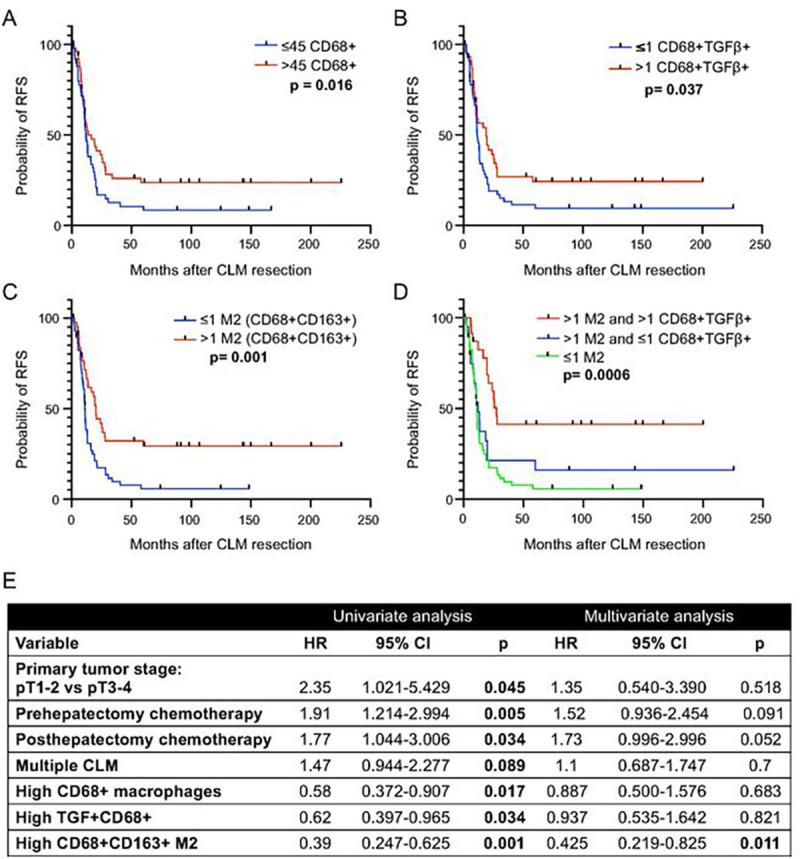
Recurrence-free survival (RFS) according to density of macrophage subtypes. **A-D,** RFS according to density of (A) total macrophages (CD68+), (B) TGFβ-expressing macrophages (CD68+TGFβ+), (C) M2 macrophages (CD68+CD163+), and (D) M2 macrophages and TGFβ-expressing macrophages. Numbers before cell phonotype descriptions are numbers of cells per mm^2^. **E,** Univariate, and multivariate Cox regression analysis for RFS (n=105). Median value used as cutoff.

In the entire study population, on univariate analysis, higher than median density (vs lower density) of the following macrophage subtypes were associated with longer RFS: CD68 + macrophages [median (range), 11.98 (0.30–225.74) months vs 11.0 (0.36–166.89) months; p = 0.016; Fig. 3A]; TGFβ-expressing macrophages [median (range), 11.61 (0.30–200.4) months vs 11.26 (0.69–225.74) months; p = 0.037; Fig. 3B]; and CD68 + CD163 + M2 macrophages [median (range), 17.44 (0.30–225.74) months vs 10.85 (1.7–148.52) months; p = 0.001; Fig. 3C)].

Expression of TGFβ on CD68 + macrophages potentiated the effect of M2 macrophages on RFS. Higher than median density of M2 macrophages in combination with higher than median density of TGFβ-expressing macrophages was associated with longer RFS than higher than median density of M2 macrophages in combination with lower than median density of TGFβ-expressing macrophages (Fig. 3D).

On multivariate analysis, only higher than median density of CD68 + CD163 + M2 macrophages was associated with longer RFS (p = 0.011, Fig. 3E).

**Figure 4:**
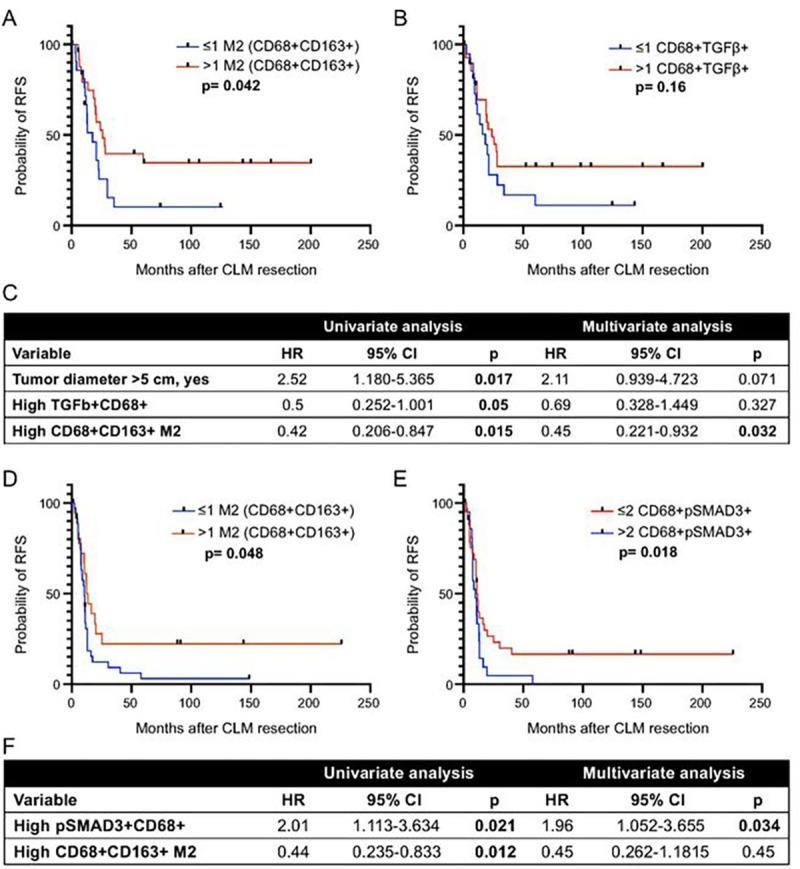
Recurrence-free survival (RFS) according to density of macrophage subtypes in patients treated with and without preoperative chemotherapy. **A-B,** RFS in patients treated without preoperative chemotherapy (n=49) according to density of (A) M2 macrophages (CD68+CD163+) and (B) TGFβ-expressing macrophages (CD68+TGFβ+). Numbers before cell phonotype descriptions are numbers of cells per mm^2^. **C,** Univariate, and multivariate Cox regression analysis for RFS in patients treated without preoperative chemotherapy (n=49). **D-E,** RFS in patients treated with preoperative chemotherapy (n=56) according to density of (A) M2 macrophages and (B) pSMAD3-expressing macrophages (CD68+pSMAD3+). Numbers before cell phonotype descriptions are numbers of cells per mm^2^. **F,** Univariate, and multivariate Cox regression analysis for RFS in patients treated with preoperative chemotherapy (n=56).

Among the patients not treated with preoperative chemotherapy, on univariate analysis, higher than median density (vs lower density) of CD68 + CD163 + M2 macrophages was associated with longer RFS [median (range), 20.36 (0.30–200.39) months vs 11.61 (1.70–124.72) months; Fig. 4A]. On multivariate analysis also, higher than median density of M2 macrophages was associated with longer RFS (p = 0.032, Fig. 4C).

Among the patients treated with preoperative chemotherapy, higher than median density (vs lower density) of CD68 + CD163 + M2 macrophages was associated with longer RFS [median (range), 12.28 (0.36–225.74) months vs 10.52 (2.39–148.52) months; p = 0.047; Fig. 4D], and higher than median density of pSMAD3-expressing macrophages was associated with shorter RFS [median (range), 9.28 (1.27–58.03) months vs 11 (0.36–225.74) months; p = 0.018, Fig. 4E]. On multivariate analysis, only higher density of pSMAD3-expressing macrophages was significant (p = 0.034; Fig. 4F).

## Discussion

Macrophages in CLM exhibit extreme heterogeneity in terms of morphology, function, and localization and hence their characterization is challenging. As macrophages exist along a polarization spectrum at any given point in time and single markers to clearly define these macrophage populations are lacking, combinations of markers are necessary to identify macrophage subsets. Our analysis revealed that the density of M2 macrophages was higher than the density of M1 macrophages in the entire cohort of CLM, was significantly lower in the CLM of patients who received preoperative chemotherapy, and predicted better RFS in patients with CLM.

Our finding of CD68 + CD163 + M2 macrophage polarization in CLM is in accordance with prior studies, which suggest that most TAMs exhibit an M2 phenotype (35–38). Wu et al. also demonstrated that MRC1 + CCL18 + M2 macrophages had higher metabolic activity in CLM than in primary colorectal cancer. These authors hypothesized that metastatic tumor cells, through expression of the ligand CD47, may recruit M2 macrophages via the SIRPA receptor (39).

Breast cancer, prostate cancer, lung cancer, and colorectal cancer have demonstrated an increase in TAM infiltration after neoadjuvant chemotherapy (40). In contrast, we observed lower density of total macrophages, M2 macrophages, and TGFβ-expressing macrophages in patients who received preoperative chemotherapy than in patients who did not receive preoperative chemotherapy. This is in accordance with a previous study in which preoperative chemotherapy led to downregulation of the metabolic status of M2 macrophages and upregulation of cytotoxic T cells (39). We did not observe any difference in the density of cytotoxic T cells between patients who did and did not receive preoperative chemotherapy.

The density of helper T cells and cytotoxic T cells was significantly higher in CLM with left-sided colonic primary tumors. Guo et al. also demonstrated that left-sided colonic primary tumors had high infiltration by cytotoxic T cells (41). We found that CLM with right-sided colonic primary tumors had higher density of TGFβ-expressing macrophages.

Macrophage polarization has been extensively studied in the literature, and the distinct roles of M1 and M2 macrophages in the TME have been broadly outlined. Briefly, M1 macrophages are considered anti-tumor due to their cytotoxic effect on tumors cells exerted via tumor necrosis factor-α and nitric oxide, whereas M2 macrophages are considered protumorigenic due to their immunosuppressive and angiogenic roles (16). Previous studies have shown that M2 macrophages are associated with worse prognosis in primary colorectal cancer (42), lung adenocarcinoma (16), ovarian cancer (43), breast cancer, and esophageal cancer (44). In contrast, Algars et al. (45) and Nagorsen et al. (46) observed that CD163-expressing M2 macrophages were associated with a better prognosis in colorectal cancer using CLEVER1/Stabillin-1. Koelzer et al. (32) and Algars et al. (45) also identified CD68 + TAMs as a positive prognostic factor in colorectal cancer. Consistent with the findings of Algers et.al, Nagrosen et. al and Koelzer et. al. in primary colorectal cancer, we found that high CD68 + CD163 + M2 macrophage density was associated with longer RFS. These results were further supported by our finding that TGFβ expression on macrophages potentiated the effect of M2 macrophages on RFS. Our finding that higher density of pSMAD3-expressing macrophages correlated with shorter RFS in the cohort with preoperative chemotherapy on both univariate and multivariate analysis (Fig. 4E–F) is interesting; this finding suggests that preoperative chemotherapy causes TME alterations in CLM leading to a significant reduction in M2 macrophage function and augments the impact of pSMAD3 expression by macrophages on survival.

A significant proportion of CD68 + macrophages did not show M1 or M2 polarization. That is likely because the state of polarization is described as the phenotype of the macrophage at a given point in time, hence TAMs exposed to multiple stimuli in the TME might exhibit phenotypes not readily classified as M1 or M2 (18–20). Multiplex immunofluorescence enabled us to incorporate a wide array of markers for macrophage characterization. Using techniques like bulk RNA sequencing or single cell sequencing would help us further delineate the macrophage subpopulations and validate our findings.

## Conclusion

In summary, ours is one of the first studies to quantify macrophages and analyze the prognostic significance of macrophage polarization in CLM using multiplex immunofluorescence. Our results provide insights into the TME of CLM and identify M2 macrophages as a predictor of better RFS. Our findings are novel; however, because of the extreme plasticity of macrophages, it will be important to explore the possibility that macrophage polarization in CLM does not strictly adhere to the M1/M2 model.

## Figures and Tables

**Table 1:** Clinicopathologic characteristics of the study population (n=105)

Characteristic	Entire cohort (n=105)	Without preoperative chemotherapy (n=49)	With preoperative chemotherapy (n=56)	P*

**Age, median (range) years**	58 (48–70)	61 (53–70)	55 (48–63)	0.031

**Sex, n (%)**				NS
Male	57 (54)	27 (55)	30 (54)	
Female	48 (46)	22 (45)	26 (46)	

**Primary tumor site, n (%)**				NS
Left colon	56 (53)	28 (57)	28 (50)	
Right colon	29 (28)	13 (227)	16 (29)	
Rectum	20 (19)	8 (16)	12 (21)	

**Lymph-node-positive primary tumor, n (%)**				NS
Yes	77(73)	34 (69)	43 (77)	
No	28 (27)	15 (31)	13 (23)	

**Extrahepatic metastasis, n (%)**				NS
Yes	10 (10)	4(8)	6 (11)	
No	95 (90)	45 (92)	50 (89)	

**Synchronous CLM, n (%)** **				<0.001
Yes	58 (55)	18 (37)	40 (71)	
No	47 (45)	31 (63)	16 (29)	

**Preoperative chemotherapy, n (%)**				
No	49 (47)	NA		
Fluoropyrimidine +oxaliplatin + bevacizumab	23 (22)		23 (41)	
Fluoropyrimidine + irinotecan + bevacizumab				
Oxaliplatin only	13 (12)		13 (23)	
Irinotecan only				
Other/more than 1 of the above regimens	9 (9)		9 (16)	
	6 (6)		6 (11)	
	5 (5		5 (9)	

**Pathologic response, n (%)** †				
Major	26 (46.4)	NA	26 (46)	
Minor	27 (48.2)		27 (48)	
Not available	3 (5.4)		3 (5)	

**Preoperative serum CEA level, median (range), ng/ml** ‡	5.4 (0.5–1349)	7.1 (0.5–1349)	2.8 (0.5–497)	0.002
**Median preoperative serum CEA level, n (%)** ‡				0.031
≤5 ng/ml	51 (49)	18 (37)	33 (59)	0.031
>5 ng/ml	54 (51)	31 (63)	23 (41)	

**Diameter of largest CLM, median (range), cm**	3.0 (0.8–15)	3.5 (1.2–15)	3 (0.8–10.5)	NS

**Number of metastatic liver nodules, n (%)**				0.009
Solitary	50 (48)	29 (59)	21 (38)	
Multiple	55(52)	20 (41)	35 (62)	

**Hepatectomy margin, n (%)**				
R1	9 (9)	3 (6)	6 (11)	
R0	96 (91)	46 (94)	50 (89)	

* Comparison between patients with and without preoperative chemotherapy.

** CLM diagnosed within 1 year after diagnosis of primary tumor.

† Major response=less than 50% residual tumor cells, minor response=more than 50% residual tumor cells

‡ At diagnosis of colorectal liver metastasis (CLM).

**Table 2:** Density of macrophage and adaptive T cell subtypes in CLM by clinical characteristics

	Diameter of largest CLM		P	Preoperative chemotherapy		P	Primary tumor location		P	Node positive primary tumor		P
**Phenotype**	**≤3 cm**	**>3 cm**		**Yes**	**No**		**Right colon**	**Left colon**		**Yes**	**No**	
**Total CD68+**	48 (2–237)	44 (12–276)	0.83	37 (2–181)	63 (14–273)	**0.002**	63 (11–273)	40 (2–236)	0.07	43 (2–273)	48 (7–236)	0.33
**CD68+CD163+ (M2)**	0 (0–36)	2 (0–97)	**0.01**	0 (0–36)	2 (0–97)	**0.03**	1 (0–97)	1 (0–23)	0.72	1 (0–97)	1 (0–36)	0.94
**CD68+TGFβ+**	1 (0–29)	1 (0–17)	0.57	1 (0–21)	2 (0–29)	**0.02**	3 (0–29)	1 (0–21)	**0.04**	1 (0–29)	1 (0–17)	0.73
**CD68+FOXP3+**	0 (0–16)	1 (0–3)	0.97	0 (0–2)	1 (0–16)	**0.001**	1 (0–2)	0 (0–16)	0.24	0 (0–16)	1 (0–3)	0.08
**CD3+CD4+**	3 (0–259)	2 (0–58)	0.66	2 (0–259)	3 (0–145)	0.49	1 (0–58)	4 (0–259)	**0.02**	3 (0–259)	2 (0–259)	0.88
**CD3+CD8+**	1 (0–49)	1 (0–26)	0.97	1 (0–49	1 (0–49)	0.17	1 (0–28)	2 (0–49)	**0.03**	1 (0–5)	1 (0–49)	0.69
**CD3+CD8+pSMAD3+**	0 (0–1)	0 (0–2)	0.47	0 (0–2)	0 (0–10)	0.15	0 (0–2)	0 (0–10)	0.44	0 (0–10)	0 (0–5)	**0.02**
**CD3+CD4+pSMAD3+**	0 (0–3)	0 (0–3)	0.41	0 (0–1)	0 (0–5)	**0.002**	0 (0–2)	0 (0–5)	0.45	0 (0–5)	0 (0–2)	0.16

a Values in table are cells/mm^2^, median (range), unless otherwise indicated.
